# The Nutritional Quality of Food Provision at UK Government-Funded Holiday Clubs: A Cross-Sectional Analysis of Energy and Nutrient Content

**DOI:** 10.3390/nu15081937

**Published:** 2023-04-18

**Authors:** Martina Vitale, Shannon Crossland, Jackie Shinwell, Paul B. Stretesky, Margaret Anne Defeyter, Iain Andrew Brownlee

**Affiliations:** 1 Department of Applied Sciences, Northumbria University, Newcastle upon Tyne NE1 8ST, UK; m.vitale@northumbria.ac.uk (M.V.); shannon.crossland@northumbria.ac.uk (S.C.); 2 Healthy Living Lab, Faculty of Health and Life Sciences, Northumbria University, Newcastle upon Tyne NE1 8ST, UK; jackie.shinwell@gmail.com (J.S.); greta.defeyter@northumbria.ac.uk (M.A.D.); 3 Department of Social Sciences, Northumbria University, Newcastle upon Tyne NE7 7YT, UK; paul.stretesky@northumbria.ac.uk

**Keywords:** diet quality score, food insecurity, food assistance, children’s food provision

## Abstract

A large proportion of children are at risk of food insecurity during school holidays in the UK. The government-funded Holiday Activities and Food (HAF) programme provides free holiday clubs offering at least one healthy meal/day to eligible children and adolescents. This study aims at evaluating the nutritional quality of food provision at HAF holiday clubs, particularly hot/cold and vegetarian/non-vegetarian meals. Menu variants (*n* = 2759) from 49 HAF holiday clubs were assessed for adherence to School Food Standards (SFS) and their notional compositional quality, which was scored utilising a novel nutrient-based meal quality index. The median adherence to SFS across all available menus was 70% (IQR 59–79%). Overall, hot variants scored statistically higher menu quality scores than cold variants for both 5–11y (92.3 (80.7–102.7) vs. 80.4 (69.3–90.6)) and 11–18y (73.5 (62.5–85.8) vs. 58.9 (50.0–70.7)) criteria. Cold and hot menu variants tended to score differentially for quality sub-components. These findings highlight areas for potential future improvement in HAF holiday club provision with a tendency for food provision to appear less ideal for attendees for those aged 11–18. Ensuring that children from low-income households have access to a healthy diet is crucial to reduce UK health inequalities.

## 1. Introduction

The UK appears to have one of the highest rates of food insecurity among children in Europe [1]. In England, 13% of children were defined as being in food insecure households in 2019/20, largely as a consequence of low family income [2]. Results from the National Child Measurement Programme have shown that English children from disadvantaged backgrounds are twice as likely to be both short in stature or obese compared to those in the least deprived backgrounds [3,4], with poor diet likely to be a major contributors to these two conditions [5,6]. Recent national screening data suggested that children and adolescents are intaking over the recommend amounts of saturated fats and more than double those of free sugars, while consuming foods low in fibre, with a further income-related gradient apparent [7]. Development of lifelong, prudent eating habits in childhood is a key determinant of health in the short and long-term: malnutrition is not only associated with impaired growth [8], depression [9] and poorer cognitive functions [10], but also with an increased risk of obesity [11] and non-communicable diseases later in life [5,12].

In order to mitigate the impact of food insecurity on the health of children and their families, measures such as free school meals and breakfast clubs are available across schools in England [13,14]. However, up to 2018, government policies to ensure that children from low-income families had access to free food did not cover the school holidays. In 2017, it was estimated that up to 3 million children and their families could become food insecure during the summer holidays in the UK [15]. There appears to have been a sharp increase (from 15.4% of pupils in 2019 to 22.5% in 2022) in the number of children attending either primary or secondary state-funded school in England who are eligible for free school meals [16], which would suggest there has also been a recent increase in need for food assistance out of the school term.

The Holiday Activities and Food (HAF) Programme pilot was launched by the Department for Education in 2018 [17]. The HAF Programme was subsequently rolled out across all 151 local authorities in England, in 2021 [18], and enables local authorities to fund holiday clubs offering food and drink options, nutritional education and stimulating activities free of charge to children eligible for benefits-related free school meals and some other children (e.g., children with special educational needs and disabilities). As a condition for the funding, holiday clubs have to provide at least one meal a day that meets the School Food Standards (SFS), which consist of food-based requirements to ensure that the food and drinks served at state-funded schools foster health and encourage positive eating habits [19]. To date, the adherence to SFS by HAF providers has not been evaluated [20,21]. Furthermore, to the authors’ knowledge, there is a lack of studies investigating the nutritional quality of the food provided. Previous research has focused on the food consumed at pre-HAF holiday clubs which showed promising, yet mixed, results [20,22,23,24]. For example, Crilley et al. (2022) found that participants followed a healthier diet overall and that what they eat was more adherent to SFS when they attended the holiday club compared to when they did not attend [20]. However, what children consumed at the lunches served at holiday clubs was not fully compliant with the SFS and on both attendance and non-attendance day the participants’ diet did not fully meet the UK Eatwell Guide recommendations [25].

Due to the complex relationship between what humans consume and health outcomes, evaluating the overall quality of either diets or meals is considered preferable to assessing single foods or nutrient content. Common practice to determine the overall nutritional quality of diets is using a priori indices, such as the Healthy Eating Index and the Mediterranean Diet Score [26]. However, indices to evaluate the quality of single meals, especially those served to children at school and similar settings, are less common [27,28]. Existing methods include components that were selected according to the issues in the target populations’ eating patterns and national nutrition guidelines [28,29]. Therefore, there is a need to develop a meal quality assessment method for meals served at HAF holiday clubs and/or schools in England. An approach that can be used is evaluating the energy and nutrient content of the meals provided instead of specific foods or drinks. This has different advantages such as being an objective measure that can be applied universally despite cultural diversity or preferences [30,31,32]. Furthermore, it can provide us with insights into what food and drinks may contribute the most to specific nutrient contents.

Out of the 420 holiday clubs included in the 2021 HAF evaluation, 30% served only cold meals (typically a packed lunch) and 41% served a combination of hot and cold meals [18], highlighting the need to assess the quality of cold and hot food provision at HAF holiday clubs. 

Due to the increased awareness of the health and environmental impact of omnivorous diets, vegetarianism and the availability of vegetarian food options are on the rise in industrialized countries [33]. The importance of reducing meat consumption is recognised in the SFS practical guide as it advises schools to encourage all pupils to have a “meat-free day” a week [19]. As such, consideration of the comparative quality of vegetarian and non-vegetarian meals served at HAF holiday clubs is also rational.

Therefore, the aims of this study are to evaluate the nutritional quality of the lunches provided at HAF holiday clubs, with a particular focus on comparing hot and cold food options and vegetarian and non-vegetarian offerings. Further from this, the adherence of available menus to School Food Standards was also assessed. 

## 2. Materials and Methods

### 2.1. Study Overview

Food provision details from 52 HAF holiday clubs were collected by Northumbria University researchers as part of an evaluation of the HAF-funded “Bring it on Brum” holiday programme that took place in Birmingham in 2021 [34]. The food provision from three of the documents collected were not analysable due to not including sufficient information. A total of 49 menus (covering 482 menu days and 2759 menu variants) were assessed. Ethical approval for this study was obtained from the Faculty of Applied Sciences’ Ethics Committee at Northumbria University (approval number: 17664, granted on 24 August 2020).

### 2.2. Assessment against School Food Standards

The current SFS includes 24 separate sub-standards which menus are expected to adhere to [19], with a binary decision made as to whether each menu had met the standard or not met the standard. Due to the nature of the available menus (which in some cases spanned less than a week), not all standards could be assessed. Provision standards that related to weekly (e.g., “One or more wholegrain varieties of starchy food each week” or “no more than 2 portions of food that have been deep-fried, batter-coated, or breadcrumb-coated, each week”) provision frequency or less (e.g., “Oily fish once or more every 3 weeks”) could not be assessed in shorter menus. The proportion of assessable standards per menu that were adhered to were reported.

### 2.3. Estimation of Nutrient Profile

The menus were analysed utilising the nutrient analysis software Microdiet version 4 (Downlee Systems, Salford, UK). The 2015 McCance and Widdowson’s integrated food composition dataset [35] was used because it focuses on the nutritional composition of the most commonly consumed foods and drinks in the UK. 

As no portion sizes, recipes and ingredients were included in any of the menus, a methodology was developed to maintain consistency and increase the results reliability. Wherever possible, the mean of recommended portions sizes for primary (5–11 years) and secondary school-age (11–18 years) children reported in the UK government guidance “Portion sizes and food groups” [36]. Alternatively, in the case of composite dishes and items for which only raw weights were specified in the government document, the portion size estimates reported by Davies et al. (2008) were used [37]. Thirdly, when HAF holiday club menus included branded items, the exact weight was retrieved by searching on one of the UK supermarket websites. When none of the above options were available, the Microdiet-recommended portion size options (e.g., “medium portion”, “one slice”) were used.

Once compositional data were processed, the lunch offerings were coded as being cold or hot, and vegetarian or non-vegetarian, to allow comparison between different menu variants. Meals were classified as cold when all the elements, even if previously cooked, were assumed to be served either cold or at room temperature. Hot menu variants mainly consisted of cooked meals served hot and that, usually, are consumed using cutlery. However, per definition of cold menu variants, when any of the elements included were hot (e.g., a sandwich served with a side of chips), the meal was classified as a hot variant. Meals were coded as vegetarian when they did not include flesh foods and, therefore, excluded all kind of meat, fish, crustaceans, molluscs and their products [38,39]. They might, however, include dairy ingredients or eggs. No distinction was made between vegan or vegetarian options as only one of the menus specified that they served no animal derived products, such as dairy, honey and eggs.

Free sugars values were subsequently calculated due to Microdiet providing an output which includes also intrinsic sugar. The 10-step methodology by Louie et al. (2015) was employed to estimate free sugars content of the different menu variants [40], with adaptation to align with the 2018 definition of free sugars by Public Health England [41]. In addition to the assumptions made to estimate free sugars content in the National Diet and Nutrition Survey (NDNS) [42], fruit salad and canned fruit were assumed to be served drained and therefore, containing 0 g of free sugars.

### 2.4. Development of the Meal Quality Index

To compare the overall nutritional quality of the different menu variants, a meal quality index was developed. The approach used the Scottish nutrient-based SFS [43], as these are the most up-to-date standards available within the UK, with updates based on the Scientific Advisory Committee on Nutrition recommendations [44,45,46].

Table 1 shows details of the meal quality index scoring approach. A total of fourteen components, including one for energy, six for macronutrients and seven for micronutrients were used to assess the overall nutritional quality of the menu variants. For each individual component, lunches could get a continuous score between 0 and 10 for a total score of 140. A higher score both in the single components and overall quality reflected greater compliance to the standards. A similar approach to scoring has been applied in previous research on the diet quality of children [20,47]. Each component was equally weighted as a combination of adequate energy, macro and micronutrients consumption is essential for good health and growth [48]. Moreover, these nutrient-based standards have already been simplified to include the most significant nutrients that determine young people and adolescents’ health, either because they are consumed excessively or not in a sufficient amount [43,49]. For these reasons, we deemed it appropriate to give each component equal weighting as categories. Different values were assigned to attendees aged 5–11 years and 11–18 years to reflect the increased nutritional requirements in adolescence [50,51].

In order to define the cut-offs for each scoring component, two different approaches were used. In the case of nutrients with a reference nutrient intake (RNI) and lower reference nutrient intake (LRNI) value (i.e., all included micronutrient categories except for sodium), the cut-off for score 0 was determined calculating the corresponding percentage of the LRNI value [51]. For example, the 5–11 years nutrient-based standard for calcium is 30% of the RNI for 7–10 years old. This value represents the cut-off for score 10 and any value greater than or equal to this would score maximally. To determine the cut-off for score 0, 30% of the iron LRNI for this specific age group was calculated and any value less than or equal to this would score 0. An upper value to indicate micronutrient toxicity was not set as this rarely occurs from food sources alone [52]. For the energy, macronutrients and sodium components, the cut-off value was determined as 50% of the age-specific standard. This approach to determine cut-offs, albeit arbitrary, has been applied in other diet quality scoring approaches [46,53,54]. In the instance of nutrients without a maximal recommended intake, such as carbohydrate, fibre and protein, any value greater than or equal to the nutrient-based standard was given a score of 10, while for a value less than or equal to 50% of the standard, the score was 0. For nutrients where recommendations exist to consume below a maximal intake (free sugars, total and saturated fats), any value greater than the nutrient-based standards was given a score of 0, while for a value less than or equal to 50% the standard, the score was 10.

The “idealness” of the energy provision was based on bidirectional proximity to the recommended, as both excess or insufficient energy provision could be considered problematic [55]. To take this eventuality into account and reduce the impact it could have on the overall quality score, both upper and lower cut-offs for energy were set. A score of 0 was again calculated taking into consideration 50% of the nutrient-based standard for energy intake.

### 2.5. Statistical Analysis

After analysing the nutritional content of the menus and assigning scores to each component according to the developed index, data were analysed with IBM SPSS Statistics version 28 (IBM Corporation, Armonk, NY, USA). Kolmogorov–Smirnov test was used to assess normality. As the test results suggested that the data were not normally distributed, Mann–Whitney U test was used to compare hot and cold variants, and vegetarian and non-vegetarian ones. A *p*-value less than 0.05 was considered statistically significant. Data have been reported as the median and interquartile range (IQR).

## 3. Results

### 3.1. School Foods Standards Adherence

Adherence to assessable SFS elements is presented in Table 2 below. A total of 167 menu options were available to analyse, of which 82/167 could be defined as vegetarian (see above) and 25/167 as cold. The median adherence to SFS across all available menus was 70% (IQR 59–79%). There were similar levels of adherence for both hot and cold menu offerings (70.6% (IQR 58.1–78.9%) vs. 70.0% (IQR 63.6–70.0%), respectively) and vegetarian versus non-vegetarian offerings (70.6% (IQR 61.1–80.7%) vs. 70.0% (IQR 57.1–77.8%), respectively). Due to the nature of the menu records received, none of the menus could be assessed against the standard “Any condiments must be limited to sachets or portions of no more than 10 g or one teaspoonfuls”. While all of the menus with adequate detail (17/17–100%) met the standard for not including salt, this was only not assessable in a further 150 menus. Of the remaining standards, the most frequently-met one was “no more than 2 portions of food that have been deep-fried; batter-coated; or breadcrumb-coated; each week” (95.6% of menus met standard), while among the least frequently met were “lower fat milk; which must be available for drinking at least once a day” (14.8%) and “one or more wholegrain varieties of starchy food each week” (22.8%). 

### 3.2. Menu Characteristics and Quality Scoring

The lunches analysed comprised 2759 menu variants (the range of lunch options on a daily basis that included possible combinations of main options, side dishes, desserts, starters and so forth). There were a total of 1320 (47.8%) cold menu variants and 1439 (52.2%) hot menu variants. There were 1288 (46.7%) menu variants that were vegetarian. For 5–11 years, the median meal quality score for all the menu variants was 87.8 (IQR = 73.6–97.8) out of a total possible score of 140. For 11–18 years variants, it was 66.3 (IQR = 55.1–78.4). 

Table 3 presents the number and percentage of menu variants meeting the nutrient-based standards [43]. Both for primary (5–11 y) and secondary school-aged (11–18) children, the nutrients that most frequently scored maximally were total fat (5–11, 83.5% and 11–18, 95.2% met, respectively), sodium (5–11, 84.3% and 11–18, 93.8%) and vitamin C (5–11, 76.0% and 73.6% for 11–18). Iron (5–11, 19.4% and 11–18, 5.3%), vitamin A (5–11, 23.2% and 11–18, 13.7%) and dietary fibre (5–11 = 21.2%, 11–18 = 4.8%) were the nutrients with the lowest proportion of menus meeting the maximal score. No single scoring component scored maximally in all the menus.

### 3.3. Hot and Cold Menu Variants

**Figure 1:**
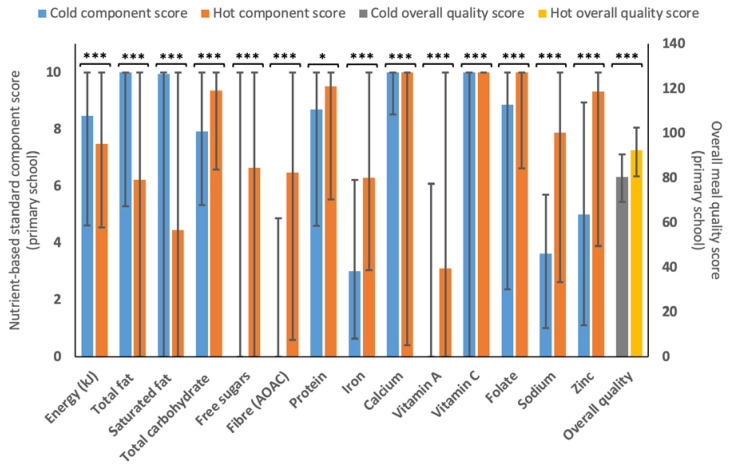
Median (IQR) for the different 5–11-year-old component scores and overall quality score for cold (*n* = 1320) and hot (*n* = 1439) menu variants. A statistically significant difference is represented by * *p* < 0.05, and *** *p* < 0.001), as determined by Mann–Whitney U test.

As shown below in Figure 1, the overall quality scores of cold (*n* = 1320) menu variants (median = 80.4, IQR = 69.3–90.6) were statistically significantly lower (*p* < 0.001) compared to hot (*n* = 1439) menu variants (median = 92.3, IQR = 80.7–102.7) for 5–11 years scores. Despite the difference in overall quality score, hot and cold menus appeared to score statistically higher across different sub-component scores. While almost all menu variants scored maximally for calcium, vitamin C and folate, hot menu variants had statistically significantly higher (*p* < 0.001) component scores for multiple elements vs. cold menus, including free sugars (6.6 (0.0–10.0) vs. 0.0 (0.0, 10.0)), fibre (6.5 (0.6–10.0) vs. 0.0 (0.0, 4.9)) iron (6.3 (3.0–10.0 vs. 3.0 (0.6 vs. 6.2)) and sodium (7.9 (2.6–10.0) vs. 3.6 (1.0–5.7)). However, the hot menu variants scored statistically significantly lower (*p* < 0.001) vs. cold menu variants for factors components including energy (7.5 (4.6–10.0) vs. 8.5 (4.6–10.0), total fat (6.2 (0.0, 10.0) vs. 10.0 (5.3–10.0)), and saturated fat (4.5 (0.0–10.0) vs. 9.9 (0.0–10.0).

**Figure 2:**
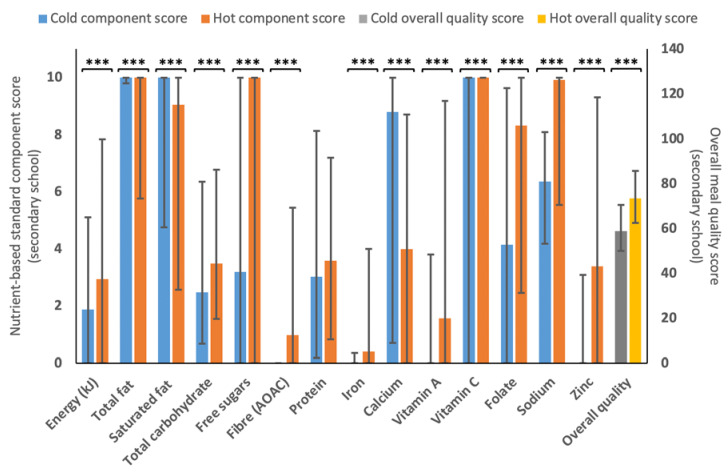
Median (IQR) for the different 11–18 years component scores and overall quality score for cold (*n* = 1320) and hot (*n* = 1439) menu variants. A statistically significant difference is represented by *** *p* < 0.001), as determined by Mann–Whitney U test.

Figure 2 shows the comparison between hot and cold meals for 11–18 years scores. Similar to 5–11 years estimates, the overall quality of the cold menu variants (58.9 (50.0–70.7)) was statistically significantly lower (*p* < 0.001) than the hot food provision one (73.5 (62.5–85.8)) and a similar overall component profile to the 5–11 years school data above (see Figure 1), with hot menus scoring statistically higher (*p* < 0.001) for free sugars (10.0 (0.0–10.0) vs. 3.2 (0.0–10.0)), fibre (1.0 (0.0–5.4) vs. 0.0 (0.0–0.0)), iron (0.4 (0.0, 4.0) vs. 0.0 (0.0–0.4)) and sodium (9.9 (5.5–10.0) vs. 6.4 (4.2–8.1)) and lower for total fat (10.0 (5.8–10.0) vs. 10.0 (9.8–10.0), saturated fat (9.0 (2.6–10.0) vs. 10.0 (4.8–10.0)) and calcium (3.4 (0.0–8.7) vs. 8.8 (0.7–10.0)).

### 3.4. Vegetarian and Non-Vegetarian Menu Variants

**Figure 3:**
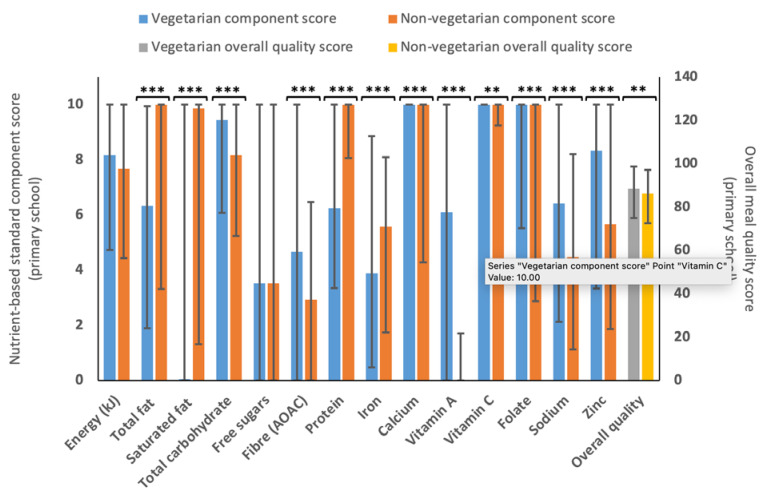
Median (IQR) for the different 5–11 years component scores and overall quality score for vegetarian (*n* = 1288) and non-vegetarian (*n* = 1471) menu variants. A statistically significant difference is represented by represented by ** *p* < 0.01 and *** *p* < 0.001, as determined by Mann–Whitney U test.

There was a small but statistically significant (*p* < 0.01) difference between the overall meal quality scores for vegetarian (*n* = 1288, median 88.6 (75.1–98.6)) and non–vegetarian (*n* = 1471, median 86.2 (72.6–97.2)) menu variants (see Figure 3). Non–vegetarian menu variants scored statistically (and considerably) higher for the saturated fat component (9.9 (1.3–10.0) vs. 0.5 (0.0–10.0)), and also scored higher (*p* < 0.001) for total fat (10.0 (3.3–10.0) vs. 6.3 (1.9–9.9)) and protein components (10.0 (8.1–10.0) vs. 6.3 (3.4–10.0)) but scored lower for most other components.

**Figure 4:**
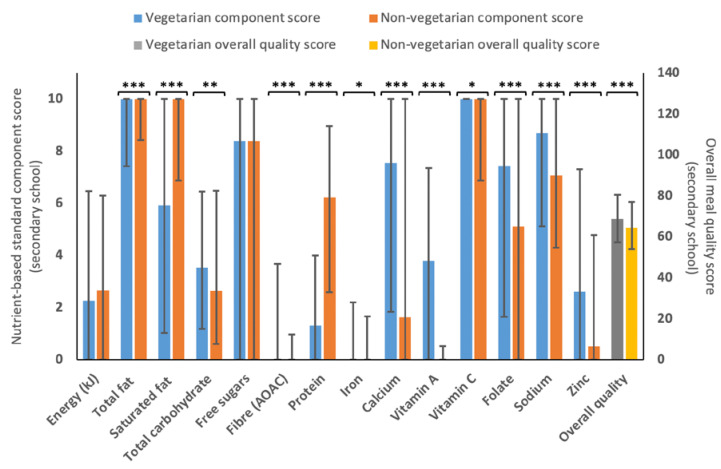
Median (IQR) for the different 11–18 years component scores and overall quality score for vegetarian (*n* = 1288) and non-vegetarian (*n* = 1471) menu variants. A statistically significant difference is represented by * *p* < 0.05, ** *p* < 0.01 and *** *p* < 0.001, as determined by Mann–Whitney U test.

The secondary school scoring also suggested that vegetarian menu variants scored statistically higher than non–vegetarian variants (68.8 (57.4–80.5) vs. 64.5 (54.1–77.1)), with higher component scores for most factors (see Figure 4) but statistically lower scores for saturated fat (5.9 (1.0–10.0) vs. 10.0 (6.9–10.0)) and protein (1.3 (0.0–4.0) vs. 6.2 (2.6–9.0)).

## 4. Discussion

This study is the first formal analysis of the nutrient and energy content of the food provision at HAF-funded holiday clubs. Furthermore, it compared hot and cold menu variants, and vegetarian and non-vegetarian meals utilising a novel meal quality index specifically developed to assess the nutritional quality of HAF holiday club lunches.

### 4.1. Adherence to School Food Standards

Despite the limitations that could affect food provision in many HAF holiday clubs, these findings suggest positive but not complete adherence to many aspects of the SFS. The findings broadly highlight a number of key areas through which HAF holiday club providers could aim to improve future provision, including making wholegrain food options and low-fat dairy and alternative sources of calcium more frequently available.

Many elements of adherence could not easily be assessed within the collected menu data, which broadly underlines the need for a standardized approach to share and evaluate menu data. Similar challenges have recently been noted in attempts to carry out evaluation of dietary quality of school food offerings [57]. 

### 4.2. Overall Meal Quality and Nutrient Content

The meal quality score findings highlight the need for improving energy and nutrient content of the food offering both for children aged 5–11 and particularly for those aged 11–18. Overall scores for secondary school criteria were generally low. This could have been influenced by the methodology used to estimate quantities. However, the mean value calculated from the government guidance [36] was often the lower end of secondary schools recommended portion sizes and, therefore, may be similar to what was served at HAF holiday clubs for the 11–18 age group. 

A low proportion of menus met the standard for fibre both for 5–11 years and 11–18 years. As only 14% of children and 4% of adolescents were meeting recommendations in the latest NDNS [7], there would appear to be a need for further focus on supporting adequate fibre intake at a national level. A 2015 systematic review showed an association between low fibre consumption and increased risk of obesity, metabolic syndrome and insulin resistance in children and adolescents [58]. Furthermore, a secondary analysis of the NDNS 2008–2014 results by Johnson et al. (2018) found that low-fibre content is one of the components of an obesogenic diet, alongside excessive calories and fat [59]. Another nutrient of concern is iron, especially for adolescents due to increased requirements in males (development of lean body mass) and females (blood loss through menstruation) [60]. Iron deficiency can lead to anaemia which is connected to both physical and mental health symptoms, such as fatigue, headaches, apathy, irritability and depression [61]. Previous research has also suggested that also iron deficiency without anaemia can affect cognitive functions and impair academic performance [62]. This could further widen the achievement gap between children from high and low socioeconomic status households, possibly determining career prospects. Between 2016 and 2019, intake of iron in 49% of British girls aged 11–18 was below the LRNI, while 9% had low iron stores and haemoglobin levels [7]. This highlights the need for public health approaches to increase iron consumption among teenage girls. Ensuring that HAF holiday club food provision meets (or ideally exceeds) the provision recommendations for this micronutrient would appear to be a low-risk and high reward strategy for supporting young people from low-income households in accessing higher dietary iron intake.

On a positive note, total fat and saturated fat content was consistently below the maximal threshold in a high proportion of menus both for primary and secondary school age standards. Consuming a higher proportion of monounsaturated and polyunsaturated fatty acids may exert a protective cardiovascular effect longer-term [63,64]. Furthermore, a 2017 meta-analysis of the association between saturated fat and health in children and adolescents found that reducing saturated fatty acid intake is associated with lower total and low-density lipoprotein cholesterol, and diastolic blood pressure [65]. A further recent meta-analysis suggested that reducing saturated fat consumption is associated with a 17% lower risk of cardiovascular events in adults [66]. Johnson et al. (2018) also found an association between socio-economic status and fat consumption: children from low-income families were more likely to follow the identified obesogenic dietary pattern that included energy dense and high-fat foods [59]. For these reasons, it is particularly positive that the HAF food provision already supports attendees in not exceeding recommendations for fat intake. However, a low percentage of menus met the energy requirements for secondary school due to not providing enough calories. HAF holiday club providers are, therefore, presented with the challenge of increasing the energy content while not compromising the components that score highly such as total and saturated fats.

### 4.3. Nutritional Quality of Hot and Cold Menu Variants

Similar to findings from previous research comparing school meals and packed lunches (prepared at the family home) [67,68,69,70,71,72,73,74,75], this study found that the overall nutritional quality of hot menu variants was statistically significantly better than cold ones (Figure 1 and Figure 2). Results are also consistent with previous findings in regard to free sugars [67,68,70,71,73], fibre [68,70,71,72,74], sodium [67,68,70,71,72,73,74] and iron [71,72] for school food provision versus cold (home-prepared) packed lunches. However, it is important to underline that these studies measured the intake of nutrients and food groups opposed to the food provided, and that the school meals could have included cold options; results may, therefore, not be comparable. Within the current study, cold options were provided by the HAF holiday clubs or through a central caterer rather than being prepared at home. Nevertheless, Pearce et al. (2013) reported that the majority of meals served at school were hot, while packed lunches consisted of cold and finger food options [72]. Contrary to previous findings [67,69,70,71,72,73,74,75], cold lunches were more adherent to standards for total and saturated fats compared to hot menu variants. Interestingly, Evans et al. (2010) found that saturated fat intake in children consuming packed lunches decreased compared to a 2004 survey [76,77] and Evans et al. (2015) did not find a difference in saturated fat intake in children consuming either a packed lunch or a school meal [68]. The authors speculated that this was due to a reduction of total and saturated fat content in snacks and prepacked foods commonly included in packed lunches. This may also apply to the nutritional composition of cold meals in the current study.

Particular attention should be placed on improving the free sugars content of cold lunches as high intakes in childhood and adolescence are strongly associated with increased incidence of dental caries and obesity [46,78,79]. Furthermore, it is important to note that the Scottish nutrient-based standard for free sugars is based on the recommendation of not exceeding 7.5% of total dietary energy intake instead of the 5% recommended by the Scientific Advisory Committee on Nutrition, as they recognised the difficulty in achieving this target in children and adolescents [43]. Thus, the current results would be even more negative if the standard is reduced to 5%. According to the latest NDNS results [7], mean free sugars consumption in children and adolescents is over 12% of total energy, underlining a need for national improvement in dietary habit. HAF programme should target limitation of free sugars within their provision in parallel with supporting nutritional educational messages targeting this and other improved health behaviours in club attendees. Based on previous research, the major contributors of free sugars in cold lunches packed for school are confectionary, cakes, yogurt and sweetened drinks [68,76]. Therefore, HAF holiday club providers should find healthier appealing alternatives to these items to include in their cold food offering.

Similar to free sugars, sodium was also consistently found to be high in packed lunches in previous research [67,68,70,71,73,74,75] and in cold menu variants in this study. High intakes of this mineral are associated with increased blood pressure and arterial stiffness in children and adolescents [80,81] with longer-term risks of hypertension and cardiovascular disease [82]. A 2022 secondary analysis of the NDNS found that sodium consumption in children from low socioeconomic status households decreased by 15% from 2008 to 2019 [83] and should be carefully monitored in school and HAF holiday club meals. Evans et al. (2010) found that the major contributors to sodium content in packed lunches for school were cheese, snacks, bread and sandwich fillings [76]. However, the authors remarked that dairy products are an important source of calcium, while sandwiches can provide protein and micronutrients. Therefore, changes should be carefully evaluated not to compromise (a) the content of these nutrients and (b) the convenience of the cold menu offering for HAF holiday club providers. Finally, even if hot menu variants scored statistically significantly better on a higher number of components, low median scores for other factors highlight the need to improve both types of menu offerings.

### 4.4. Nutritional Quality of Vegetarian and Non-Vegetarian Menu Variants

Vegetarian menu variants had consistently higher meal quality scores. This finding underlines the idea that well-planned vegetarian diets are an appropriate and nutritionally positive option [84]. However, non-vegetarian meals scored higher for total fat, saturated fat and protein (among others). Previous research about school food provision in France reported no difference in the quality of vegetarian and non-vegetarian meals [85,86], while another found that replacing meat or fish with a vegetarian alternative worsened the nutritionally quality [87]. There would also appear to be a need to improve total vitamin A provision in both vegetarian and non-vegetarian offerings. Previous studies have noted similar issues in US school food provision [88].

Perhaps surprisingly, non-vegetarian menu variants scored statistically significantly better for total and saturated fat. Vegetarian meals were found to be high in saturated fat in previous research [86,89], due to the important contribution of eggs and dairy in this (ovo-lactovegetarian) dietary pattern. HAF holiday club providers could evaluate how to integrate other vegetarian options (e.g., legumes and pulses into the menus), which would be expected to both improve fat content and have other benefits on overall menu nutrient provision [90]. However, inclusion of a variety of protein sources across the menus will help to avoid issues with nutrient imbalance and protein quality [91]. Furthermore, a study by Seves et al. (2017) found that in the scenario where all meat and dairy were substituted with plant-based alternatives, saturated fat and sodium would decrease but so would important micronutrients (zinc, vitamins A, B1, B12 and calcium) [92]. Alternative vegetarian protein sources may also include highly processed foods that may be more acceptable due to mimicking taste and texture of animal products but could be less healthy or nutrient-dense than meat and pulses [92,93]. Knowledge of food preparation and interaction within nutrients is crucial to plan and improve a vegetarian diet [84,94].

The above highlights the complexity of planning food provision, especially for children following more restrictive dietary practices. Due to the critical contribution of food systems to climate change [95] and the consistent evidence that well-planned vegetarian dietary habits tend to be more environmentally positive [85,92], such considerationss should be encouraged in government-funded settings.

### 4.5. Strengths and Limitations

The main strength of the current work is the dual approach to assessment of menu quality. The authors believe that these approaches provide a platform from which to develop future approaches to analyse the quality of both HAF holiday club and school food provision. While these are designed with UK metrics to define scoring criteria, similar approaches could easily be developed in other regions of the world where food- and nutrient-based guidelines may differ.

Within the current study, the food-based evaluation may be more realistic under the operational limitations of menu analysis and allow evaluation of provision across the whole day. However, the nutrient-based evaluations help to highlight less obvious limitations of food provision, in particular, the differential quality patterns noted in hot and cold lunch offerings. While the current study was based on a preliminary evaluation of HAF programmes in a single area, the depth of data is underlined by the large range of menu options and variants possible from a relatively small number of original menus. The novel approach to meal quality scoring developed in this study was based on the most up-to-date and evidence-based requirements for the target age groups attending HAF holiday clubs.

Nevertheless, the present study has some limitations that are important to acknowledge. First of all, weight and food composition were an estimate as menus did not include this information. However, utilising existing evidence meant that it was possible to estimate portion sizes that are likely to be served to the age groups attending holiday clubs [36,37]. The authors cannot exclude the potential risk that portion sizes for 5–11-year-olds were overestimated, while those for 11–18-year-olds were underestimated because the selected weight was the mean between primary and secondary school recommended servings. However, the calculated portion weight was often shared across recommendations for the two age groups, meaning that the estimated portion size may at least overlap with possible portion sizes served at HAF holiday clubs. This highlights a need for more accurate estimation of portion sizes served at HAF clubs for future evaluation.

Another important limitation is that the cold menu variants only came mainly from a relatively small proportion of the menus, meaning that the certainty of median quality estimates is somewhat limited. Despite the low number of holiday clubs providing cold meals, the multiple combinations they offered meant that the number of cold menu variants was still considerably high. Finally, this study included the food provision from a HAF programmes running in a single geographic area. This may therefore not be more widely representative of HAF food offerings. There is the potential that the location of different clubs (e.g., urban or rural areas) and the cultural background of attendees (e.g., eating habits affected by religion) may impact the way in which clubs are able to provide food. Nonetheless, these preliminary findings are, to the authors’ knowledge, the first in-depth evaluation of the nutritional quality of food served at the new HAF-governed holiday club programmes.

### 4.6. Future Work and Impact of Findings

Due to the limitations of this research, future studies could measure weights of portions sizes in HAF holiday club settings and analyse the food provision from a wider range of HAF-funded holiday programmes. Furthermore, it is crucial to assess if the food offered is consumed by holiday club attendees as this would determine the extent to which the HAF programme is improving the diet of children and adolescents. To the authors’ knowledge, no research has investigated HAF holiday club meals uptake. Therefore, future studies could quantify the number of attendees that consumes packed lunches brought from home and how nutritionally adequate they are. As the concept of nutritional quality is complex and multi-faceted [96], it is important that future HAF holiday club food provision evaluations include further parameters in addition to energy and nutrient content, such as variety and food groups. Evaluating if HAF holiday clubs’ food provision comply with food-based SFS could be a useful “first-pass” approach to define the need for further improvement. Evaluation of the menus of a wider range of HAF holiday clubs would allow better inference and understanding of how SFS adherence may (or may not) align with nutrient-based quality scores.

A 2022 study found that intake of ultra-processed food in British school meals comprised 61% of a primary school lunch total energy content and 70.1% of a secondary school one [97]. Therefore, future research could focus on measuring the amount of ultraprocessed food offered in HAF holiday club menus. Lastly, secondary analysis of this dataset or future evaluations could assess which food or drink source contributes the most to each nutrient content. Translating nutrient information to what food and drinks should be increased or limited would support HAF holiday club providers with limited nutrition knowledge in improving their food offering and the government to provide specific guidance.

With an estimated cost of over GBP 6 billion a year, consequences of a poor diet are a major financial burden for the NHS [98]. It is clear that ensuring that young people consume a healthy diet is of paramount importance. Approaches like the HAF programme can prevent children from gaining weight and developing non-communicable diseases later in life with a positive impact on health expenditure. Multiple stakeholders will be impacted by this research. First of all, the findings highlight the need for the government and local authorities to provide professional guidance, particularly around the cold food offering and specific nutrients that were consistently low across the menus. Due to the important role that food provision covers in the HAF programme, the government should consider implementing a strong “monitoring, evaluation, accountability and learning” process, rather than relying on HAF holiday clubs to self-rate food provision. Secondly, this study contains some easy-to-interpret information, such as possible major contributors to free sugars content in cold lunches, that could be immediately applied by HAF holiday club providers to improve their food offering. Thirdly, the novel approach developed in this study to evaluate energy, nutrient and overall meal quality could be employed by researchers to assess not only HAF holiday club lunches but also those offered in schools. Finally, and most importantly, this research could impact HAF holiday club attendees’ health and well-being if the nutritional quality of the meals is improved as a consequence of the findings.

## 5. Conclusions

In conclusion, the suboptimal overall meal quality scores highlight the need to improve the energy and nutrient content of HAF-funded holiday club food provision, particularly to meet the increased nutritional requirements of 11–18-year-olds. In the menus analysed, improving the food offerings for older attendees with very different energy and nutrient requirements should be a priority. There is a need to improve fibre, iron, free sugar and sodium content of the many of the food offerings to target improvement overall notions of ideal lunches. There is a future need to engage with HAF holiday club providers in developing a rational approach to evaluate menus in the future, to ensure it is both feasible and fit for purpose. Based on the current findings, we recommend that the Department for Education and local authorities should provide guidance to decrease free sugars and sodium in cold menu variants, improve micronutrients content of non-vegetarian options and reduce saturated fats in vegetarian meals without compromising the overall meal quality. Due to the complexity of nutrition, the government should consider involving professionals to plan menu variants that meet the nutritional needs of children and adolescents attending HAF-funded holiday clubs.

## Figures and Tables

**Table 1:** Table showing the meal quality index * cut-offs for the lowest (score 0) and highest (score 10) component scores for primary (5–11) and secondary school-aged (11–18) children.

Component	Minimum (0) Scoring Criteria		Maximum (10) Scoring Criteria	
	Primary (5–11 years)	Secondary (11–18 years)	Primary (5–11 years)	Secondary (11–18 years)
Energy (kJ)	≤1082.5 kJ or ≥3247.5 kJ ^b^	≤1557 kJ or ≥4671 kJ ^b^	2165 kJ (±325 kJ) ^a^	3114 kJ (±467 kJ) ^a^
Fat	20.1 g ^a^	29 g ^a^	≤10 g ^b^	≤14.5 g ^b^
Saturated fat	6.3 g ^a^	9.1 g ^a^	≤3.15 g ^b^	≤4.5 g ^b^
Total CHO	≤34.5 g ^b^	≤49.6 g ^b^	≥69.1 g ^a^	≥99.3 g ^a^
Free sugars	10.4 g ^a^	14.9 g ^a^	≤5.2 g ^b^	≤7.4 g ^b^
Fibre	≤3 g ^b^	≤4.5 g ^b^	≥6 g ^a^	≥9 g ^a^
Protein	≤9.7 g ^b^	≤13.9 g ^b^	≥19.4 g ^a^	≥27.9 g ^a^
Iron	≤1.6 mg ^d^	≤2.4 mg ^c^	≥3 mg ^a^	≥4.4 mg ^a^
Calcium	≤97.5 mg ^c^	≤144 mg ^c^	≥165 mg ^a^	≥300 mg ^a^
Vitamin A	≤75 µg ^c^	≤80 µg ^c^	≥150 µg ^a^	≥187 µg ^a^
Vitamin C	≤2.4 mg ^c^	≤3 mg ^c^	≥9 mg/d ^a^	≥11 mg ^a^
Folate	≤22.5 µg ^c^	≤30 µg ^c^	≥45 µg ^a^	≥60 µg ^a^
Sodium (mg)	686 mg ^a^	824 mg ^a^	≤343 mg ^b^	≤412 mg ^b^
Zinc	≤1.2 mg ^c^	≤1.65 mg ^c^	≥2.1 mg ^a^	≥2.8 mg ^a^

* The developed meal quality index is based on the nutrient-based school food standards for Scotland [43]. ^a^ Same values as in nutrient-based school food standards (Scotland) [43]. ^b^ 50% higher or lower than nutrient-based school food standards (Scotland) [43]. ^c^ 30% of lower reference nutrient intake [51] for the age group considered in the school food standards (Scotland) [43]. ^d^ 35% of lower reference nutrient intake [51] for the age group considered in the school food standards (Scotland) [43].

**Table 2:** Menu adherence to each element of the School Food Standards.

School Food Standard	% Adherence (*n*/Total Assessable)	% Unassessable (of 167)
A1. ≥ 1 portions of starchy foods per day	95.2 (159/167)	0 (0)
A2. ≥ 3 different starchy foods each week *	79.8 (99/124)	25.7 (43
A3. ≥ 1 wholegrain varieties each week	22.8 (23/101)	39.5 (66)
A4. Starchy food cooked in fat/oil ≤ 2 days each week	87.7 (107/122)	26.9 (45)
A5. Bread (with no added fat or oil) must be available every day	43.3 (61/141)	15.6 (26)
1. ≥ 1 portion of vegetables or salad as an accompaniment every day	50.3 (84/167)	0.0 (0)
B2. ≥ 1 portions of fruit every day	72.5 (121/167)	0.0 (0)
B3. Dessert containing ≥ 50% fruit 2+ times per week	75.2 (88/117)	29.9 (50)
B4. ≥ 3 different fruits and ≥ 3 different vegetables each week	64.5 (71/110)	34.1 (57)
C1. A portion of food from this group every day	53.3 (88/165)	1.2 (2)
C2. Lower fat milk must be available for drinking at least once a day during school (HAF holiday club) hours	14.8 (9/61)	63.5 (106)
D1. A portion of food from this group every day	64.0 (103/161)	3.6 (6)
D2. A portion of meat or poultry on ≥ 3 days each week? *	60.6 (43/71)	57.5 (96)
D3. Oily fish ≥ 1 every three weeks	34.0 (17/40)	70.1 (117)
D4. For vegetarians, a portion of non-dairy protein on ≥ 3 days each week *	61.9 (39/63)	62.3 (104)
D5. A meat or poultry product (manufactured or homemade, and meeting the legal requirements) ≤ 1 week *	71.0 (49/69)	58.7 (98)
E1. ≤ 2 portions of food that have been deep-fried or coated per week	96.0 (119/124)	25.7 (43)
E2. ≤ 2 portions of food which include pastry each week? *	96.0 (120/125)	25.1 (42)
E3. No snacks, except nuts, seeds, vegetables and fruit with no added salt, sugar or fat	72.7 (117/161)	3.6 (6)
E4. No confectionery, chocolate or chocolate coated products	64.6 (104/161)	3.6 (6)
E5. Desserts, cakes and biscuits are allowed at lunchtime. They must not contain any confectionery	57.1 (80/140)	16.2 (27)
E6. Salt must not be available to add to food after it has been cooked **	100.0 (17/17)	89.8 (150)
E7. Any condiments must be limited to sachets or portions of no more than 10 g or one teaspoonful *	0 (0/167)	100 (167)
F1. One of the permitted drink options **	93.7 (59/63)	62.3 (104)

School Food Standards defined further in [19,56]. * Primary school-age (5–11) cut-off (≤once per week) used to define meeting standard. ** Permitted drinks options are: (a) plain water (still or carbonated) (b) lower fat milk or lactose reduced milk, fruit or vegetable juice (max 150 mL), (c) plain soya, rice or oat drinks enriched with calcium; plain fermented milk (e.g., yoghurt) drinks, (d) combinations of fruit or vegetable juice with plain water (still or carbonated, with no added sugars or honey), (e) combinations of fruit juice and lower fat milk or plain yoghurt, plain soya, rice or oat drinks enriched with calcium; cocoa and lower fat milk; flavoured lower fat milk, all with less than 5% added sugars or honey, (f) tea, coffee, or hot chocolate, (g) combination drinks are limited to a portion size of 330 mL (and may contain added vitamins or minerals), and no more than 150 mL of fruit or vegetable juice (which must be at least 45% fruit or vegetable juice).

**Table 3:** Number and percentage of HAF menu variants (*n* = 2759) meeting the nutrient-based standards for 5–11 years and 11–18 years [43].

Nutrient-Based Standard	Primary School-Age (5–11) *n* (%)	Secondary School-Age (11–18) *n* (%)
Energy (kJ)	948 (34.4)	241 (8.7)
Total fat	2303 (83.5)	2627 (95.2)
Saturated fat	1776 (64.4)	2357 (85.4)
Total carbohydrate	1186 (43.0)	161 (5.8)
Free sugars	1562 (56.6)	1799 (65.2)
Fibre (AOAC)	584 (21.2)	133 (4.8)
Protein	1284 (46.5)	280 (10.2)
Iron	535 (19.4)	145 (5.3)
Calcium	1762 (63.9)	889 (32.2)
Vitamin A	639 (23.2)	377 (13.7)
Vitamin C	2098 (76.0)	2031 (73.6)
Folate	1529 (55.4)	881 (31.9)
Sodium	2325 (84.3)	2587 (93.8)
Zinc	971 (35.2)	362 (13.1)

## Data Availability

The data presented in this study are available on request from the corresponding author. The data are not publicly available due to ethical and privacy concerns.
